# Internalization of ferromagnetic nanowires by different living cells

**DOI:** 10.1186/1477-3155-4-9

**Published:** 2006-09-05

**Authors:** Adriele Prina-Mello, Zhu Diao, John Michael David Coey

**Affiliations:** 1 Centre for Research on Adaptive Nanostructures and Nanodevices (CRANN) and School of Physics. Trinity College Dublin, Dublin 2, Ireland

## Abstract

The ability of living cells, either adherent or suspended, to internalize nickel nanowires is demonstrated for MC3T3-E1, UMR106-tumour and Marrow-Stromal cells. Nanowires were produced by electrodeposition, 20 μm long and 200 nm in diameter. Cell separation and manipulation was achieved for the three cell types. Applied magnetic field successfully oriented the internalized nanowires but no clear anisotropy is induced on the adherent cells. Nanowires tend to bind to cytoplasm metalloproteins and trigger lysosome reorganization around the nucleus. This work demonstrates the applications of nanowires in adherent and suspended cells for cell separation and manipulation, and further explore into their role in nanobiotechnology.

## Background

Ferromagnetic nanoparticles have found widespread uses in modern biology and medicine [1-3]. They are used as contrast agents for magnetic resonance imaging [4], localized radio-frequency heating [5] and applying mechanical stress in magnetic tweezers [6]. Applications include functionalized labeling [3] for separation [7], drug delivery [8], imaging and detection [9]. Most current cell labeling techniques use one of two approaches: (1) attaching magnetic nanoparticles to the cell surface or (2) internalizing the nanoparticles by fluid phase endocytosis [10].

Studies of ferromagnetic nanowires are much less advanced. The advantages over the use of nanowires instead of nanoparticles are related to the favourable geometrical anisotropy, the increased surface to volume ratio and dipolar magnetic properties linked to the nanowire shape. Furthermore by using magnetic nanowires with large permanent magnetic moments it is possible to increase the range and effectiveness of such magnetic interactions to respond to the weak fields at a distance from external magnets.

Techniques are well developed to produce these wires by electrodeposition into porous anodized alumina templates in milligram quantities, and it is possible to tailor their length by changing the deposition time, and their diameter by choosing a suitable template [13,14,16]. The wire composition may be uniform or variable along the length of the wire by varying the conditions of electrodeposition. The ability to code information and spatially-varying functionality into the nanowires, as well as their shape anisotropy, means that they have a potentially greater range of useful applications than nanoparticles.

Recently it has been shown by Reich and co-workers [7,11,12] that nanowires can be internalized by immortalized fibroblasts, and can be used in biotechnological applications. There, the optimization and yield of magnetic nanowires was carried out as an alternative to magnetic particle separation system and as a cell sorting and positioning system.

The orientation and manipulation of adherent cells by internalized magnetic nanowires has not been extensively exploited [7]. Information in literature is restricted to just one cell type, NIH 3T3 mouse fibroblast cells [7,11,12].

Here we investigate a range of cells – undifferentiated, differentiated and tumour cells – and show how they can absorb nickel nanowires in the adherent and suspended states. We investigate the possibility of alignment of these cells in an external magnetic field, with a view towards the alignment of cells for preferential tissue regeneration and bone growth. Finally, we also speculate about the cellular internalization process and activated cytoplasmic mechanism.

## Results

### Magnetic properties of nanowires

**Figure 1:**
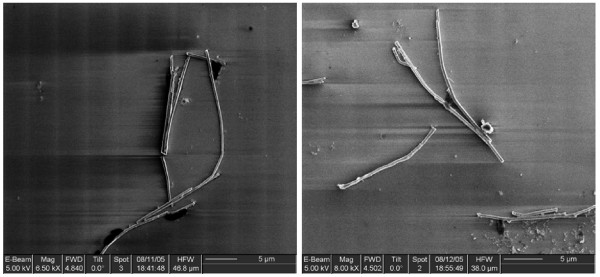
Scanning electron micrograph of nickel nanowires after dissolving the alumina template.

**Figure 2:**
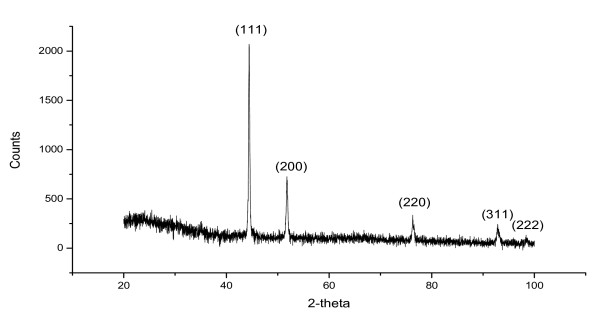
X-ray diffraction pattern of the nickel nanowire powder.

**Figure 3:**
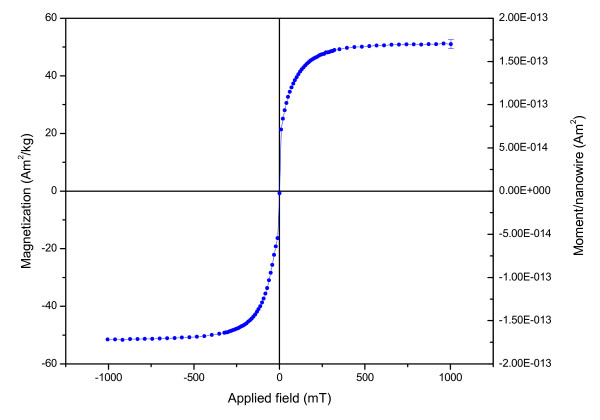
Room temperature magnetization curve for nickel nanowires produced by electrodeposition in porous alumina templates.

The X-ray diffraction pattern of the SEM imaged nanowires (Fig. 1), showed a single Ni phase pattern (Fig. 2) (powder diffraction, X-rays wavelength of CuKa (λ = 1.5418 Å), 2-θ values in agreement with JCPDS No:04-0850). The room-temperature magnetization curve of the nickel nanowires is shown in Fig. 3. The saturation magnetization is 50.7 Am^2^kg^-1^, which is significantly less than the value for bulk nickel (55.4 Am^2^kg^-1^). The EDAX analysis indicated an O/Ni ratio of 1:15, hence it seems likely that the surface of the wires is coated with a layer of nickel oxide which is approximately 3–4 nm thick, with a certain amount of Al_2_O_3_. The absence of NiO and Al_2_O_3 _peaks in X-ray diffraction pattern can be explained by the amorphous nature of the oxides. The magnetic moment, *m *of a typical wire is 1.6 × 10^-13 ^A m^2^.

### Magnetic separations

The presence of the nanowires made it easy to separate the cells containing a wire from those which did not. The separation experiment was carried out as described above with the MC3T3-E1 and UMR-106 cells. As a result, more than 70 % and 60 % separation purity, defined as the cells captured with nanowires divided by the total number of captured cells, was achieved for MC3T3-E1 cells and UMR cells respectively, which is consistent with the work by Hultgren et. al. [7]. The separated cells were then replated and cultured at physiological condition till confluency, up to 3 days, and then divided for further experimental separation. All the primary cells and cell lineages with internalised magnetic nanowires responded with a total cell survival higher than 95 % (live/death viability/cytotoxicity kit, Molecular Probes, USA) up to 5 days after separation.

### Nanowires in cell culture. MC3T3-E1 osteoblast cells

**Figure 4:**
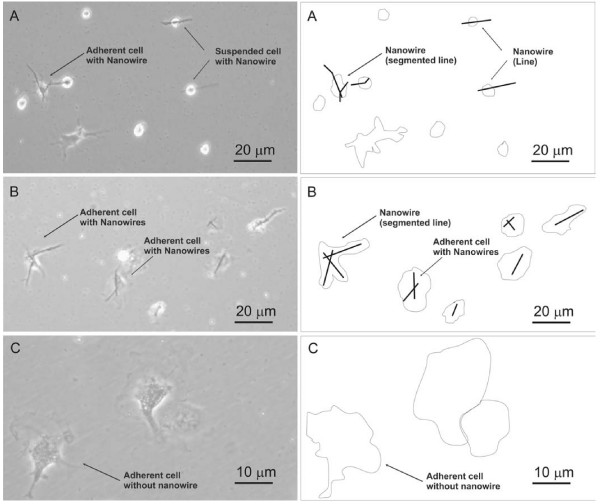
Micrographs of MC3T3-E1 osteoblasts showing: A) pre-cell separation with non-adherent cells (mag. ×20), B) adherent cells with internalized nanowires (mag. ×20), C) cells without nanowires (control, mag. ×40). All the optical micrographs have associated schematic diagrams to highlight the main features of interest for each image.

**Figure 5:**
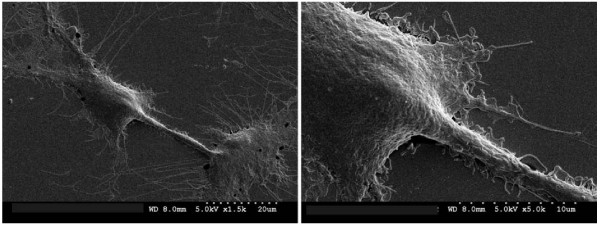
Scanning electron micrograph of adherent MC3T3-E1 osteoblasts showing internalized nickel nanowire. The nanowire drives tethering between cells.

From the magnetically-separated cell-nanowire colonies single cell manipulation and imaging was achieved by scanning electron and fluorescent microscopy. Figure 4 shows the osteoblast cell line with each cell containing one or more wires. An image taken with the scanning electron microscope (Fig 5) shows a nanowire inside the cell. There, the left cell extends out towards the right cell and uses the wire main axis as an alignment guidance. At the same time, due to the difference in mechanical properties between nanowire and cell, the nanowire introduces an anisotropical stiffening contribution to the cellular internal structures.

**Figure 6:**
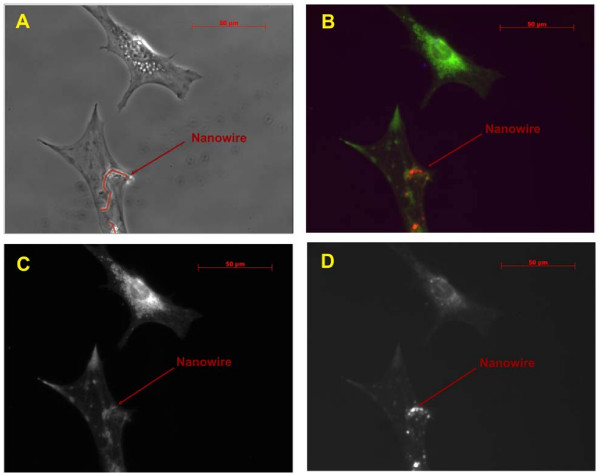
Micrographs of two MC3T3-E1 osteoblast cells after overnight incubation with and without internalized nickel nanowires (mag. ×40). A) Phase contrast picture of both osteoblast adherent cells. (Internalized nanowire highlighted in red) B) Living cell staining: double staining with Lyso-tracker (red) and Mito-tracker (green). The two cells show a completely different cytoplasm internal activity. C) Mitochondria only staining on both cells with localized activity of the cell with nanowires around lamellipodia and nanowires loci. D) Lysosomes only staining. Lysosomes are particularly localized around the bent nanowires.

To investigate this mechanical re-arrangement in cell cytoplasm evoked by nanowire internalization, fluorescent microscopy was used. To show this difference in cell activity, MC3T3-E1 osteoblast single cell with and without internalised nanowire were co-cultured and stained with living mitochondria and lysosome fluorescent trackers. Figure 6 clearly show the difference in cell organelles distribution. While the normal cell has a clear mitochondrial fluorescent staining due to normal cell activity but no lysosome staining, the cell with a nanowire has a completely different response.

A combined staining for lysosomes and mitochondria localized around the nanowire indicates that the internal organelles inside the cytoplasm respond to the nanowire. In parallel to that localized mitochondrial staining also shows lamellipodia extensions due to cell tethering and re-alignment. This could be associated with a nanowire-induced cell stiffening response.

### UMR106 osteosarcoma cells

**Figure 7:**
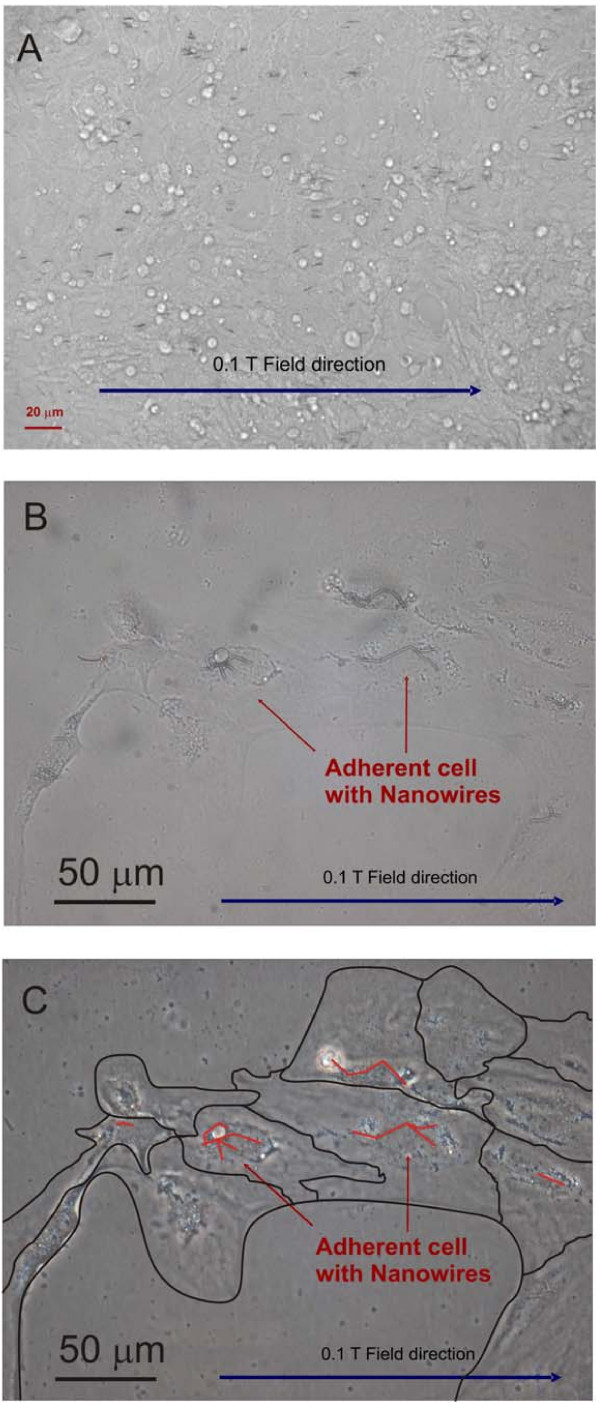
A) Confluent UMR-106 tumor cells: alignment experiment. Cell with nanowires exposed to 0.1 T for 18 hrs and then cultured to confluent layer for 5 days. The nanowires in this case are about 10 μm long. B) Colony of UMR-106 cells containing nickel nanowires (bright field contrast micrograph, mag. ×40). C) Colony of UMR-106 cells containing nickel nanowires (phase contrast micrograph, mag. ×40). Diagram of cell structures and internalized wires have been highlighted under the same z-focus level.

We showed that after separation and replating to confluence half of the cells inside each field of view contain wires and they are clearly distinguishable, Figure 7. Here, it was important to show that UMR106 cells have the same size as the nanowires and that we succeeded in the internalization process. This was achieved by the external magnetic field stimulation of the nanowires and subsequent orientation of small colonies of UMR106, as shown in Fig. 7B,7C.

To extend this finding to a larger statistical population, 5 batches, and 100 fields of view were analyzed for each group studied. In total several thousand cells were counted. The quantitative results achieved for the osteosarcoma cells were similar to those reported for the primary MSCs.

### Marrow stromal cells

**Figure 8:**
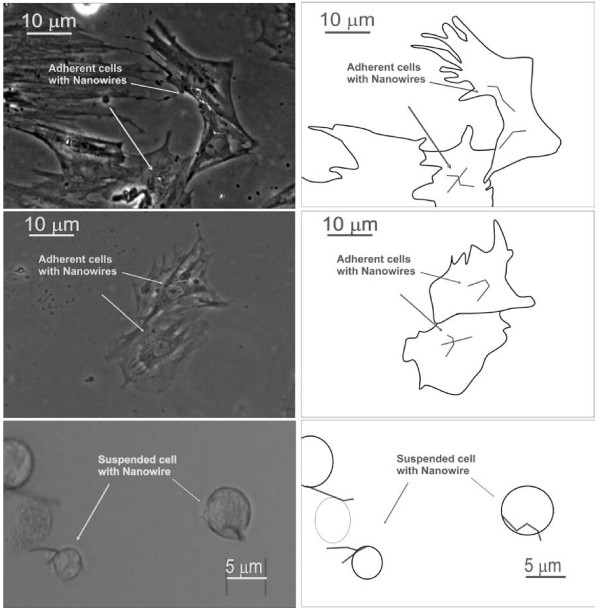
Micrographs of marrow stromal cells with internalized nickel nanowires A) Colony adherent onto plastic dishes, B) Cell-cell interaction before magnetic alignment exposure, and C) MS cells floating in DMEM medium. Phase contrast images (mag. ×40). All the optical micrographs have associated schematic diagrams to highlight the main features of interest for each image.

Marrow Stromal (MS) cells were successfully separated and isolated in small colonies to further investigate the internal cell cytoskeleton re-arrangement. Small colonies and single MS cells were stimulated and recorded by phase microscopy. Figure 8 shows both adherent cells and floating cells with internalized nickel nanowires.

The MS cells were time lapse imaged for a total period of 12-hours under phase contrast microscope, which was set so that there was a progressive heating of the culture medium, which led successively to cell detachment from the substrate and subsequent programmed cell death [see Additional file 1]. The video shows floating cells ingesting a nanowire, as well as adherent cells containing a nanowire detaching from the substrate, and eventually undergoing programmed cell death. The dead cells subsequently release part of the cytoplasm and the internalised nanowire.

**Figure 9:**
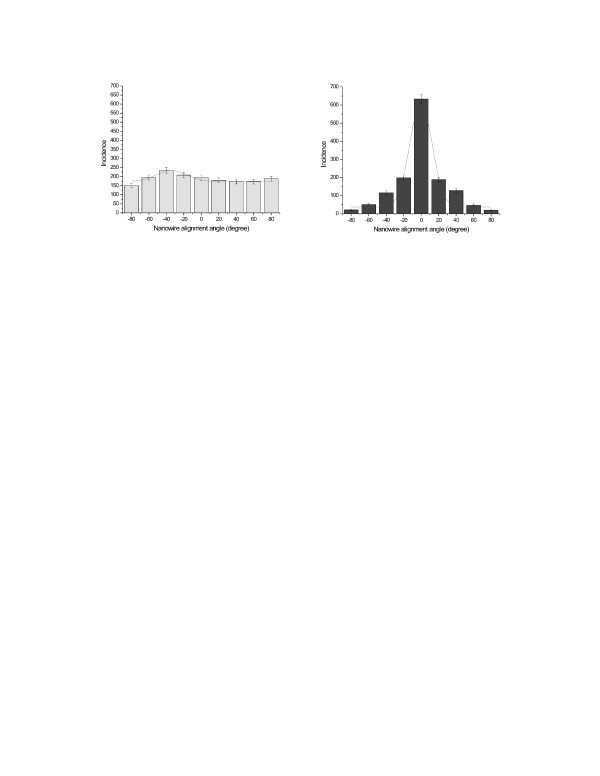
Angular distributions of nickel nanowire orientation in marrow stromal cells in zero field (left), and after alignment in a 100 mT applied field for 18 h (right).

**Figure 10:**
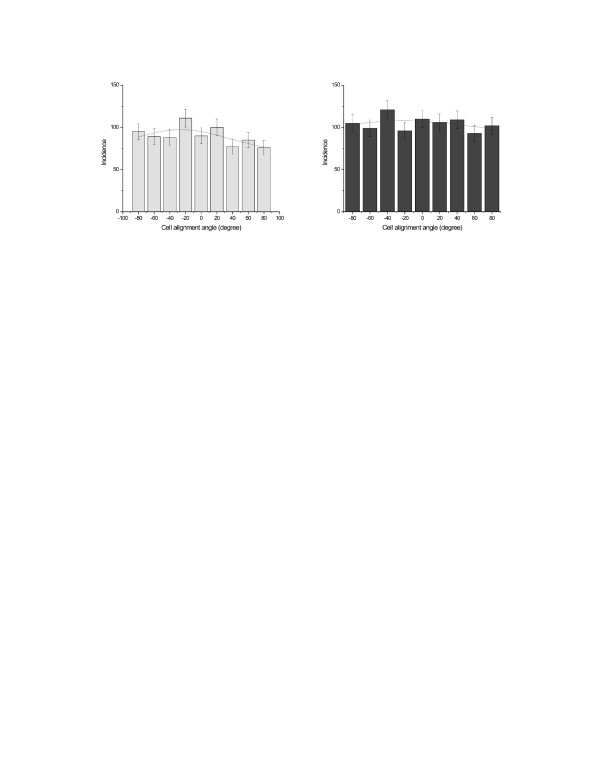
Angular distributions of marrow stromal cells, with nanowire, orientation and cell morphology in zero field (left), and after alignment in a 100 mT applied field for 18 h (right).

The alignment experiments of primary MS cells were aimed to achieve orientation in an external magnetic field. This was achieved for MS cells in suspension where manipulation and cell-to-cell bridging was reported [see Additional file 2]. These were carried out in parallel to the UMR106 osteosarcoma cells. As mentioned for the UMR106, 5 batches and 100 fields of view were analyzed for each group of study. In total, several thousand cells were counted. The histograms shown in Figure 9 and 10, plot the angular distribution of the nanowires and cells, in the absence and presence of the 100 mT magnetic field. The data are fitted to a Gaussian distribution.

While it can be seen that there is a clear orientation of the wires in the applied field, the orientation of the cells themselves is barely significant. Several experiments were carried out to investigate the extent of cell alignment versus the anisotropic orientation of the wires.

## Discussion

The viability of the three types of cells is not significantly compromised by the internalization of the nickel nanowires. The cytotoxicity of metallic nickel has been partially discussed in previous studies [7,17]. There we assessed the cytotoxicity by live/death assay. Cells were culture up to 5 days with a survival rate of 95 %. A critical factor for this survival rate may be the presence of the 3–4 nm oxide layer, inferred from the magnetization measurements and the EDAX analysis. Magnetic properties and quality of the characterized electrodeposited nanowires in this study are comparable with previous studies [14,16]. Here, we found that the internalization by cells of nanowires with the same length of suspended MC3T3-E1 was promoted by the activation of the plasma membrane receptors associated with the cytoplasm metalloproteins. This was translated into a preferential accumulation of the nanowires close to the cell nucleus membrane, as shown in Fig. 6. The localized activation of lysosomes and mitochondria around the cell nucleus support our results.

In previous works it was highlighted that nickel is an essential structural component of the metalloproteins. Nickel can enter the cell via various routes. Ni^2+ ^ions may enter the cell utilizing the divalent cation receptor [15] or via the Mg^2+ ^channel, which are both situated in plasma membrane. In other work it was shown that insoluble nickel microparticles can be phagocytised by the cell. The phagocytosis of nickel-containing compounds was enhanced by their crystalline nature, negative surface energy, and appropriate particle size (2–4 μm) [17]. In that study, it was also found that nickel particles fused with lysosomes and were localized around the cell nucleus although speculation were made over the possible mutagenicity of the insoluble nickel content.

Cell separation via nanowires can be done for all these types of cells, which indicates the versatility of the method investigated here for any type of cell manipulation [11]. In this study the multi-lineage approach to the cell separation was implemented for the three types of adherent cells (see Fig. 4, 7 and 8). The wire here drives the anisotropic adhesion of the cells, as shown in Fig. 4. The extension to primary cell then confirmed the effectiveness of the internalization of the nanowires and highlights the difference between adherent and suspended cells (Fig. 8). A future direction is to exploit the functionalization of ferromagnetic nanowires in order to actively control cell-nanowire interaction. This may lead to possible commercial application in biotechnology such as cell purification, cell isolation, cell detection [18], single cell probing or small volume drug delivery [19].

The orientation of the wires that have been ingested by the cells is quite clear for suspended cells, where the orientational effect is to be expected, due to the lack of cell adhesion or tethering of the actin filaments. There, a small magnetic field (approx 10 mT) applied to the magnetic nanowires is sufficient to re-orient the floating cells towards the direction of the applied field, since the magnetic energy of the cell containing a nanowire *mB *≈ 1.6·10^-15 ^J is much greater than *kT *≈ 4·10^-21 ^J. This principle has been used for fibroblast cells (NIH3T3 cells) by Reich and co-workers [7,11,12]. There, the optimization and yield of magnetic nanowires was demonstrated as an alternative to magnetic particle separation system and as a cell sorting and positioning system, with a focus on microfluidic applications such parallel plate flow chamber for cell sorting.

On the other hand, the orientation and manipulation of adherent cells by anexternal magnetic field using internalized magnetic nanowires for the alignment of groups of cells may have great potential in tissue differentiation and regeneration [20]. The orientation of different cell lines such as immortalized and primary cells would create a preferential co-culture in the presence of external magnetic stimulus. The main goal here was focused on the orientation of a tumor cell line (UMR 106) and primary cells harvested from adult mouse (marrow stromal cells, MSCs). In both cases the orientation force was induced by the anisotropic alignment of the internalised magnetic nanowires. This was qualitatively investigated for UMR106 (Fig. 7) and quantitatively for MSC, as shown in Fig 9 and 10.

The magnetic torque applied to each cell during the alignment process is approximately *mB *≈ 1.6·10^-14 ^Nm. The clear alignment of the nanowires indicates the resistance of the rearrangement of the nanowires is smaller than the cell adhesion force, calculated for human bone marrow stromal cell (HBMSC) (E_HBMSC _= 3 kPa, cell diam. ≅ 130 μm) [21].

This is shown in the two histograms reported in Fig. 9. However, in this study we found that there is little correlation between the cell morphology and the orientation of the nanowires, as by comparison of quantitative histograms in figure 9 and figure 10. It is probably either the consequence of the cell-nanowire interaction due to the internalization process or a considerably large mechano-chemical adhesion force exerted by the cells to the surface.

Therefore from the results of this study we can conclude not only that we have successfully achieved the internalization of nanowires on different cell lines (such as MC3T3-E1 osteoblast, Marrow Stromal and UMR106 osteosarcoma cells) but also that the interaction of the nickel nanowires with each cell cytoplasm leads to the internal reorganization of those organelles affected by the nanowires, such as lysosomes and metalloproteins.

## Conclusion

Magnetic nanowires open new perspectives for the manipulation, identification and counting of many types of living cells. The viability of different cell types, here shown, is encouraging and opens a new window for the application of ferromagnetic nanowires. Therefore, integration of different magnetic materials inside cells open new prospective in tissue engineering and nanobiotechnology.

## Methods

### Fabrication of nanowires

Nickel nanowires were grown in alumina membranes (Whatman, UK). The membranes used were 20 mm in diameter and 60 μm thick, with 200 nm parallel-pores spaced by 300 nm. Firstly a gold electrode was deposited on the back of the membrane [13]. Then, nickel was deposited into the pores by electroplating from a NiSO_4 _bath, at a potential of -0.9 to -1.0 V relative to an Ag, AgCl/KCl reference electrode [13]. The nickel wires were then separated from the membrane by dissolving the alumina membrane in 3 M·l^-1 ^NaOH [14]. The nanowires were then characterized by scanning electron microscopy (Fig. 1), X-ray diffraction (Fig. 2), X-ray fluorescence and vibrating-sample magnetometry (VSM).

### Cell type and culture environment

In this study three cell lines were used: primary cells from adult rat marrow stromal cells (MSC), MC3T3-E1 osteoblasts cell line and rat osteosarcoma cells (UMR106) (ATCC, USA). Every cell type used in this study was cultured under a different environment and culture medium, in line with the specific cell-culture requirements.

Primary MSCs were cultured and incubated at 37°C, 5 % CO_2 _and 95 % relative humidity, to confluency with DMEM supplemented with 2 % penicillin/streptomycin (Gibco, UK), 10 % foetal Bovine Serum (FBS; Gibco, UK), 0.5 % l-Glutamine (Gibco, UK), 0.5 % Glutamax (Gibco, UK), and 1 % non-essential amino acids (Gibco, UK).

For the UMR 106 cell line, all samples were cultured and incubated as above with DMEM (30-2002 ATCC, USA) supplemented with 10 % foetal bovine serum, 100 U/ml penicillin and 100 μg/ml streptomycin.

Whereas, MC3T3-E1 were cultured in alpha minimum essential medium (α-MEM: Sigma-Aldrich, UK) containing 10 % foetal bovine serum (FBS; Gibco, UK), 100 U/ml penicillin and 100 μm/ml streptomycin, 2 mM L-glutamine (Gibco, UK) and 0.5 % 100 mM sodium pyruvate (Sigma-Aldrich, UK). The osteoblasts were kept in an incubator at 37°C in a 95 % air 5 % CO_2 _environment. Subculture was routinely performed at ~80 % confluence, using 0.25 % trypsin.

### Internalization of wires

Nickel nanowires were firstly washed 5 times in deionized water (Ω <= 30 M ohm) and rinsed in different PBS baths. The nanowires were then stabilized for 2 hours into the specifics cell culture medium used for each cell line with a density of 10^11 ^particles/ml and then dispersed by 5 minutes ultrasonic agitation immediately prior to addition. Cell medium-nanowire solution was then added at a final concentration of 10^6 ^particles/ml to adhering and suspended cells. Adhering cells were incubated for 30 minutes to promote the internalization of the wires and then incubated overnight. Suspended cells were incubated for 30 minutes and then exposed to magnetic field. After each magnetic exposure, cells on coverslips were subsequently fixed or stained as required for the imaging technique adopted.

Each experiment was repeated several times as part of a larger study to address the extent of nanowires internalization and cell yield.

### Immunofluorescence staining and imaging

To characterize the ingestion of nanowires into the cells several imaging and immunofluorescence techniques were used. These were optimized for each cell type to examine the interaction between the cells and the nanowires. In this study all the three cell types were selectively stained for tracking active mitochondria and acidic organelles in live cells to highlight the cell activity during internalization.

The cell staining and preparation was carried out by staining live cells for cell tracking. In this study two cytoskeleton markers were Myto- and Lyso-tracker (Molecular Probes, USA). In brief, after having prepared a 1 mM stock solution of each tracker, a final concentration diluted in the respective growth medium (described above) of 50 nM for Myto-tracker and 50 nM for Lyso-tracker was respectively used to stain samples. Single and double staining on adherent and suspended live cell were carried out for 30 min for Lyso-tracker and 3 min for Myto-tracker; samples were then washed with fresh warm medium and imaging were taken. Subsequent, to live imaging the stained cells were permeabilised and fixed in 4.1 % paraformaldehyde in PBS for 30 min. These concentrations were kept as low as possible in order to reduce possible overloading and artefacts.

Phase contrast, bright-field and time lapse imaging microscopy were carried out with the use of a Zeiss inverted microscope (Zeiss Axiovert 200 M, Germany) connected to a cooled CCD camera (Zeiss Axiocam HRm, Germany).

### SEM preparation and imaging

SEM imaging was carried out on adherent MC3T3-E1 and MSC cells with internalized wires. All cell samples were prepared by fixing each glass coverslip slide with 3 % Glutaraldehyde in 0.1 M Sodium Cacodylate buffer (pH 7.2). The primary fixation was carried out for 1 hour at room temperature. Samples were then washed 6 times for 1 hour with either 0.05 M Phosphate or 0.1 M Cacodylate buffer to remove any un-reacted Glutaraldehyde from the samples before rinse and dehydration was carried out. The samples were then quickly rinsed with 50 % ethanol; dehydrated with 10 %, 30 %, 50 %, 70 %, and 95 % ethanol for 10 min each; and dehydrated with 100 % ethanol twice for 15 min each. The samples were then gold sputter-coated for high resolution imaging.

### Internalization process imaging

The ingestion of nanowires into the cells was examined by time lapse contrast microscopy. It was possible to examine the interaction of the cells with the nanowires by programming the Zeiss microscope to acquire an image every 15 minutes for 18 hrs (as reported in the supplemental material video). There, the light was programmed to heat the medium so that both adherent and floating cells could be examined.

### Magnetic separation and alignment of cell-nanowire population

The cell separation set-up was made of two Nd_2_Fe_14_B permanent magnets of 10 mm diameter and 15 mm length in antiparallel configuration at opposite sides of a 10 ml falcon tube for cell suspension. The surface magnetic field of the magnets of 0.6 T was measured by hall sensor gaussmeter (Hirst, UK). The two magnets fixed on either side of the tube together produced a magnetic field gradient up to 100 T/m.

Cells cultured with nanowires were dissociated with warmed trypsin plus EDTA4Na solution (Sigma-Aldrich, UK) for 5 min. From each full batch half was put into the magnetic separation set-up, and the other half was replated with 3 ml of fresh medium to estimate the amount of cell binding with nanowires pre-separation.

The magnetic separation was carried out for 5 minutes in order to obtain the complete sedimentation of those cells without nanowires. These last, after separation were removed and plated in 3 ml fresh medium in a new Petri dish. After having removed the magnets, the cells with nanowires were dispersed into 3 ml of fresh medium and replated in a new Petri dish.

Complete cell adhesion was achieved in less then 1 h of incubation at physiological conditions for all the batches and then cell counting was carried out in 2–3 rounds to minimize systematic and operator errors. Images were captured using an Olympus BX41 microscope (Japan) with magnifications of 10×, 20×, and 40× connected to a cooled CCD camera (Image II, USA). All images were acquired and stored by using Analysis imaging software (Analysis, Germany).

For the nanowire and cell alignment test, a uniform magnetic field of 100 mT was applied by a cylindrical Halbach permanent magnet made of segments of Nd_2_Fe_14_B (Magnetic Solutions, Ireland). The inner and outer radii of the magnet were 106 and 156 mm, respectively. Cells with Ni nanowires were co-cultured overnight before the test. The duration of the magnetic field exposure was chosen to be 1 hr, 24 hrs or 48 hrs inside an incubator at physiological conditions (T = 37°C, CO_2 _= 5 % and RH = 95 %). Subsequently, images were taken under phase contrast as described above. The degree of alignment of the wires and cell morphology to the direction of the field was conducted by using Scion Image software (Scion Corporation, USA). Both cell and nanowire counting were carried out under blind conditions to reduce systematic errors.

## Competing interests

The author(s) declare that they have no competing interests.

## Authors' contributions

APM and ZD did most of the experiments and data analysis. APM and JMDC coordinated the experiments and drafted the manuscript. All authors read and approved the final manuscript.

## Supplementary Material

Additional File 1 Cell-death induced by localized nanowire heat. Video showing the behavior of marrow stromal cells in the presence of nickel nanowires. The duration of the video corresponds to a period of 18 hours. The medium, initially at 37°C is heated at a rate of around 2°C h^-1 ^which leads to cell death. Click here for file

Additional File 2 Cell manipulation: bridging between cells. Video showing marrow stromal cell-to-cell bridging with nickel nanowire magnetic manipulation. The duration of the video corresponds to a period of 1 hour in which cells have been manipulated in cell culture medium at physiological conditions. Click here for file
